# The use of quality information by general practitioners: does it alter choices? A randomized clustered study

**DOI:** 10.1186/1471-2296-14-95

**Published:** 2013-07-08

**Authors:** David Ikkersheim, Xander Koolman

**Affiliations:** 1 KPMG Plexus, Breukelen, The Netherlands; 2 FALW, VU University, Amsterdam, The Netherlands

## Abstract

**Background:**

Following the introduction of elements of managed competition in the Netherlands in 2006, General Practitioners (GPs) and patients were given the role to select treatment hospital using public quality information. In this study we investigate to what extent hospital preferences of GP’s are affected by performance indicators on medical effectiveness and patient experiences. We selected three conditions: breast cancer, cataract surgery, and hip and knee replacement.

**Methods:**

After an inquiry 26 out of 226 GPs in the region signed up to participate in our study. After a 2:1 randomization, we analyzed the referral patterns in the region using three groups of GPs: GPs (n=17) who used the report cards and received personal clarification, GPs that signed up for the study but were assigned to the control group (n=9), and the GPs outside the study (n=200).

We conducted a difference in differences analysis where the choice for a particular hospital was the dependent variable and time (2009 or 2010), the sum score of the CQI, the sum score of the PI’s and dummy variables for the individual hospitals were used as independent variables.

**Results:**

The analysis of the conditions together and cataract surgery and hip and knee replacement separately, showed no significant relationships between the scores on the report cards and the referral patterns of the GPs. For breast cancer our analysis revealed that GPs in the intervention group refer 1.0% (p=0.01) more to hospitals that score one percent point better on the indicators for medical effectiveness.

**Conclusion:**

Our study provides empirical evidence that GP referral patterns were unaffected by the available quality information, except for the outcome indicators for breast cancer care that were presented. This finding was surprising since our study was designed to identify changes in hospital preference (1) amongst the most motivated GP’s, (2) that received personal clarification of the performance indicators, and (3) selected indicators/conditions from a large set of indicators that they believed were most important. This finding may differ when quality information is based on outcome indicators with a clinically relevant difference, as shown by our indicators for breast cancer treatment. We believe that the current set of (largely process) hospital quality indicators do not serve the GP’s information needs and consequently quality plays little role in the selection of hospitals for treatment.

## Background

Health care reform is widespread among Western countries in search of more efficient health care provision [1]. While countries with private payers like the Netherlands introduced a form of managed competition, other countries with a public payer system like the United Kingdom (UK) introduced elements of provider competition [2-4]. Regardless of health care system, recent health care reforms stimulate providers to compete for the benefit of the patients [3,5]. To achieve this goal provider quality needs to be transparent [6,7]. Public reporting of provider quality can enable quality improvement at the provider and system level. Providers may use the information to improve processes and results [8]. Patients, payers, and referring professionals such as general practitioners (GPs) may use the information to select providers, and thus shifting capacity towards the high quality providers [8].

Much is expected from GPs, who know their patients’ conditions and circumstances, are able to evaluate quality information and know their patients place great trust in their advice [7]. Consequently, policy makers in different countries are strengthening the position of GPs to allow them to guide the patient to the appropriate hospital [5,9]. For instance, within proposed NHS reform the GP consortia will commission the majority of care for their patients [10]. Also in the US initiatives such as ‘Medical Homes’ are introduced, where primary care physicians are expected to take on the responsibility for coordination of care, which includes referring people to the right provider [11,12]. In the Netherlands, quality information about hospitals became public, to allow patients and GPs to choose hospitals based on objective indicators [5,13].

Previous studies show that patients may experience difficulties interpreting quality information on report cards [14]. This may be one of the factors that providers that perform well on report card metrics do not attract more patients even if they outperform other providers on public metrics for consecutive years [15]. GPs know which outcomes are important for patients and what processes may lead to these outcomes. Consequently, they are well equipped for judging the meaning and relevance of quality information. In addition, research shows that in the Dutch context GPs have significant influence in directing patients: 68% of patients who searched for information to select a hospital, state that they determined their choice for a hospital based on the advice of their GP [16]. This percentage is likely to be higher for patients that do not seek information. This puts GPs in the driving seat, and the success or failure of competition on quality depends largely upon the extent to which GPs use quality information to refer patients.

In this study we investigate to what extent GPs are influenced in their hospital choice by using report cards with quality indicators on medical effectiveness and patient experiences for the conditions: breast cancer, cataract surgery, hip and knee replacement when referring patients to hospitals.

## Methods

### Recruitment, randomization and report cards

To recruit GPs for our study in September 2009 we sent all 226 GPs in the region Eindhoven a letter, followed-up by phone calls. Based on this enquiry 26 GPs signed up for our study. Based on publicly available quality indicators we drafted report cards with both patient experience and medical effectiveness indicators. For patient experiences we used the Customer Quality Indexes (CQI), which is partly based on the Consumer Assessment of Healthcare Providers and Systems) [13,17-19]. In the medical effectiveness domain we selected a shortlist of indicators, from the institute ‘Transparent Care’. This institute followed from a nationwide initiative to develop, measure and publish process and outcome indicators that mostly originated from scientific literature [20].

**Figure 1:**
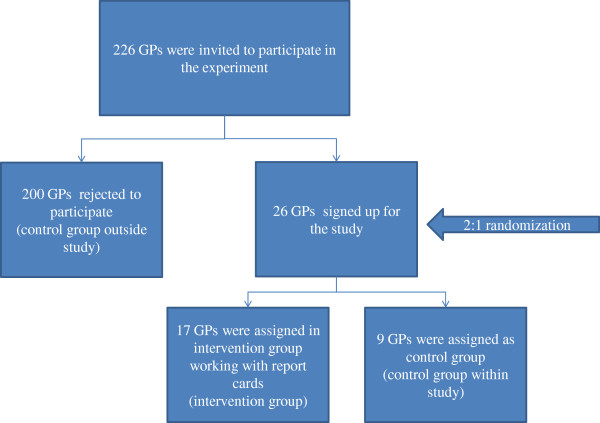
Study recruitment and randomization diagram.

We randomly assigned GPs in the intervention group and the control group in a 2:1 proportion. 17 GPs worked with report cards and 9 GPs continued working without report cards and functioned as control group. In addition we used the data of the non-participation GPs (n=200) as a control group outside our study (Figure 1).

By doing so we set up a randomized clustered trial in the south of the Netherlands around the city Eindhoven, that consists of 217,000 inhabitants and the metropolitan area has nearly 750,000 inhabitants [21]. Within this area four general hospitals are located within a diameter of 15 kilometers, that all provide treatment of breast cancer, cataract surgery, hip and knee replacement.

We then discussed the drafts of the report cards with the GPs of the intervention group to identify the most relevant indicators from the GPs’ perspective. These indicators were selected for this study. During this discussion, it turned out that the quality information presented for each hospital was new for the GPs and did not match their informed opinion. GPs selected only outcome and process indicators (rather than structure indicators) and preferred an equal distribution between indicators for patient experience and medical effectiveness on the report cards. The final paper report cards were sent to the intervention group accompanied by background information and scientific references. All 17 GPs in the intervention group received a one hour instruction on how to interpret the report cards. The report cards were presented as plasticized papers for convenience. The actual use and value of the report cards was determined by the individual GP, the only request was that the report card be discussed with the patient prior to the referral to the hospital. The used report cards are shown in Additional file 1: Table S1, Additional file 2: Table S2 and Additional file 3: Table S3.

### Data collection and statistical analyses referral patterns

Nearly all^a^ GPs in the region Eindhoven use the digital application ‘Health Domain’ to electronically refer patients to hospitals [22]. To monitor referral patterns of GPs we used data of Health Domain of participating (n=26) and non-participating GPs (n=200) for the 12 month period of the study (year 2010) and the 12 months prior to the study (year 2009).

Our database consists of all individual referrals from GPs to hospitals, where every individual referral is a record in our database. We linked the appropriate sum score of the indicators of patient experience (CQI) and medical effectiveness (performance indicators (PI’s) as separate categories per referral in the database, such that a higher sum score relate to better performance of a hospital.^b^ In addition, we related waiting times (in number of days to the first outpatient visit) per condition per hospital per week to the unique referrals using the waiting list registry that is available in the application ‘Health Domain’ , that is weekly updated by the hospitals themselves.

To analyze the effects of our intervention we used a difference in differences analysis. We used this technique to estimate changes in the referral patterns due to the introduction of the report cards in 2010 in the intervention group. While the differences between the intervention group and the control group within the study may be confounded (due to chance), we expect the difference in changes in referral patterns over time to be comparable. This means that our statistical method corrects for potentially unobserved confounding differences and cluster effects of practices in the control and treatment groups that are fixed over time [23].

For the difference in difference approach we used a logistic regression. In our database every individual referral is a row and we quadrupled every referral, simulating the choice each patient/GP have had between the four hospitals in the region. We then labeled the actual choice for the hospital of referral with a ‘1’ and the three hospitals that were not chosen with a ‘0’. These variables 0 and 1 were the dependent variable in our regression.

We used the following variables as independent variables in our regression:

– The sum score of the CQI and PI’s per hospital per condition;

– The interaction terms between the group (intervention, control group within study) and the sum score of the CQI and PI’s per hospital per condition, each for the years 2009 and 2010, so in total eight terms:

○ one term for the general effect (in years 2009 and 2010) of the CQ in the intervention group;

○ one term for the general effect (in years 2009 and 2010) of the CQ in the control group within the study;

○ one term for the general effect (in years 2009 and 2010) of the PI’s in the intervention group;

○ one term for the general effect (in years 2009 and 2010) of the PI’s in the control group within the study;

○ one term for the specific effect in 2010 (year with report cards) of the CQ in the intervention group (see Results section);

○ one term for the specific effect in 2010 (year with report cards) of the CQ in the control group within the study (see Results section);

○ one term for the specific effect in 2010 (year with report cards) of the PI’s in the intervention group (see Results section);

○ one term for the specific effect in 2010 (year with report cards) of the PI’s in the control group within the study (see Results section).

– The waiting time per condition per hospital per week of the referral;

– We corrected for cluster effects of hospitals using dummy variables per individual hospital.

For the significant coefficients we transformed these coefficients of the logit regression into percentages using a marginal effect function in Stata (mfx, predict (p) – see Results section).

### Interviews with GPs in intervention group

After the study period we held semi-structured 30 minute interview with all 17 GPs in the intervention group to qualitatively assess:

– The ease of the use of report cards

– How GPs interpretated the report cards (where some hospitals delivering better quality of care in their view?)

– To discuss the expected impact of the GPs on their referrals (did they alter their referral patterns based on the report cards?)

After these interviews a short report per interview was made and sent for review to the GPs (interviews were not recorded). We read all these reports and summarized our findings in the Result section of this study.

### Ethical approval

Ethical approval for this study was given by the nationwide ethics committee (Centrale Commissie Mensgebonden Onderzoek (CCMO)) as part of receiving the funding by ZonMw, the Dutch organization for health research and development.

### Consent

Written informed consent was obtained from the patient for publication of this report.

## Results

### Baseline data

In Table 1 the characteristics of the GPs who participated in the study are given.

**Table 1:** Characteristics groups of GPs

	**Intervention group**	**Control group within study**
**N**	17	9
**% male**	41%	44%
**% in urban area (in city Eindhoven)**	35%	33%

In Table 2 the total number of referrals to hospitals per GP group are displayed, per condition per year. The percentages shown, are percentages based on the total number of referrals for that specific condition, group and year (number in cell divided by number in column ‘Total’ on the right side of Table 2).

**Table 2:** Baseline characteristics referral patterns

	**Queens hospital**	**Essex hospital**	**Violet Hill hospital**	**Flower hospital**	**Total**
Breast cancer					
**Control group outside study (n=200 GPs) – 2009**	348 (24%)	406 (28%)	475 (33%)	229 (16%)	1458
**Control group outside study (n=200 GPs) - 2010**	440 (25%)	494 (28%)	513 (29%)	296 (17%)	1743
**Intervention group (n=17 GPs) – 2009**	13 (19%)	18 (27%)	13 (19%)	23 (34%)	67
**Intervention group (n=17 GPs) - 2010**	24 (32%)	13 (17%)	25 (33%)	14 (18%)	76
**Control group within study (n=9 GPs) – 2009**	4 (27%)	6 (40%)	2 (13%)	3 (20%)	15
**Control group within study (n=9 GPs) - 2010**	2 (17%)	3 (25%)	5 (42%)	2 (17%)	12
Cataract surgery					
**Control group outside study (n=200 GPs)- 2009**	249 (27%)	225 (24%)	250 (27%)	209 (22%)	933
**Control group outside study (n=200 GPs) - 2010**	341 (28%)	328 (27%)	307 (25%)	242 (20%)	1218
**Intervention group (n=17 GPs) – 2009**	29 (55%)	2 (4%)	9 (17%)	13 (25%)	53
**Intervention group (n=17 GPs) - 2010**	28 (41%)	7 (10%)	11 (16%)	22 (32%)	68
**Control group within study (n=9 GPs) - 2009**	3 (21%)	0 (0%)	2 (14%)	9 (64%)	14
**Control group within study (n=9 GPs) -2010**	2 (14%)	0 (0%)	7 (50%)	5 (36%)	14
Hip and knee replacement					
**Control group outside study (n=200 GPs) - 2009**	1443 (22%)	1752 (26%)	2121 (32%)	1387 (21%)	6703
**Control group outside study (n=200 GPs) - 2010**	1598 (22%)	2040 (28%)	1921 (26%)	1764 (24%)	7323
**Intervention group (n=17 GPs) - 2009**	85 (30%)	41 (14%)	82 (29%)	75 (27%)	283
**Intervention group (n=17 GPs) - 2010**	83 (28%)	61 (20%)	74 (25%)	82 (27%)	300
**Control group within study (n=9 GPs) - 2009**	36 (32%)	26 (23%)	33 (29%)	17 (15%)	112
**Control group within study (n=9 GPs) - 2010**	47 (38%)	18 (15%)	26 (21%)	33 (27%)	124
Total all referrals, all years, all groups					**20516**

### Impact report cards on choices

Our regression analysis showed no significant results comparing the intervention group with the non intervention group. In Table 3 the outcome of the logistic regression on the referral patterns are shown, comparing the 26 GPs who signed up for the study with the 200 GPs who did not sign up. As mentioned in the Method section, in the table the four parameters that compare the intervention group with the control group outside the study are displayed per condition and for the total. For breast cancer specifically our analysis shows that GPs in the intervention group refer 1.0% (p=0.01) more to hospitals that score one percent point better on the indicators for medical effectiveness. In addition, also for breast cancer specifically, GPs refer 1.4% (p=0.01) less to hospitals that score one point higher on the CQI. The analysis of all conditions together and cataract surgery and hip and knee replacement separately, show no significant relationships between the scores on the report cards and the referrals of the GPs.

**Table 3:** Logistic regression on impact report cards on referral patterns of GPs

**Compared to control group outside study year 2010**		** Coefficient**	**P- value**	** Confidence intervals**	
Total (all conditions)	CQI intervention group year 2010	−0.001	0.73	−0.0083	−0.0058
	CQI control group within study year 2010	−0.000	0.99	−0.0090	0.0089
	PI intervention group year 2010	0.000	0.94	−0.0008	0.0008
	PI control group within study year 2010	0.000	0.92	−0.0007	0.0007
Breast cancer	**CQI intervention group year 2010**	**−0.066 (-1.4%)**	**0.01**	**−0.1184**	**−0.0141**
	CQI control group within study year 2010	−0.011	0.72	−0.0700	0.0485
	**PI intervention group year 2010**	**0.046 (1.0%)**	**0.01**	**0.0092**	**0.0830**
	PI control group within study year 2010	0.007	0.72	−0.0311	0.0449
Cataract surgery	CQI intervention group year 2010	−0.188	0.74	−1.3234	0.9483
	CQI control group within study year 2010	−0.228	0.64	-.1.2003	0.7438
	PI intervention group year 2010	0.005	0.74	−0.0250	0.0348
	PI control group within study year 2010	0.006	0.64	−0.0196	0.0316
Hip and Knee replacement	CQI intervention group year 2010	0.207	0.18	−0.1012	0.5157
	CQI control group within study year 2010	0.232	0.26	−0.1755	0.6387
	PI intervention group year 2010	−0.010	0.19	−0.0256	0.0052
	PI control group within study year 2010	−0.011	0.26	−0.0306	0.0084

### Interviews with GPs

The GPs unanimously stated that - in general- there was a lack of differentiation between the scores on the report cards, or that the scores were contradictory. For instance for the report card regarding cataract surgery the Essex hospital performs well on the patient experiences, but it performs rather poor on the domain of medical effectiveness.

For the report card regarding hip and knee replacements, there is a lack of differentiation for the patient experiences and GPs stated that the differences in the indicators for medical effectiveness are rather small and some scores are unlikely to be so good (Flower hospital). In addition, they mentioned the fact that indicators on the report cards give *process* rather than *outcome* information, what limits the potential impact of the report cards even more so. In contrast, the majority of GPs said that the report card for breast cancer was an exception, with more differentiating and also more relevant indicators as the indicator *“% breast cancer operations where radical surgery is achieved during first surgery”* was considered a rather meaningful measure, where confounding was unlikely. In addition, the scores on this indicator per hospital were supported by the other indicator on the report card: the volume of breast cancer patients per surgeon per year, where many studies have shown a positive volume outcome relationship for this condition [24].

During the interviews it became clear that aspects other than quality information on report cards had impact on the GPs’ referral to a hospital. These aspects include: personal preferences of the GP, communication of specialists after discharge with the GP, consultation options of medical specialists and whether the GPs knows the medical specialist who he is referring to in person.

GPs stated that they were unsure whether the report cards altered their referral patterns and also mentioned that taken the time to thoroughly discuss the report cards with patients was about 5 minutes, which is fairly long as a standard consult takes about 10 minutes in a Dutch GP practice.

## Discussion

In many countries policy reforms are implemented that rely on GPs to guide patients to the ‘best’ hospitals using publicly available quality information. If a success, these reforms enhance the medical quality and patient centeredness of these health care systems, especially in areas where multiple providers operate. In this study we show the effect of report card use by GPs in referring their patients to the hospital under optimal conditions. In contrast to prior studies we do not use surveys of patients or GPs, but use actual referral data [25,26]. Given the training and experience of GPs, GPs should be well equipped to understand, interpret and use the quality information wisely. In addition, patients rely on their GP for their hospital choice. Given these circumstances we expect that if the current quality information would have the potential to alter choices of patients for hospitals, our study should bring this to light.

Our analyses show mixed results regarding the impact of report cards. The overall analyses did not show significant alteration in referral patterns of GPs in the intervention group compared to the control group within and outside the study in line with previous studies [27,28]. It should be noted, that given the rather small number of referrals in the intervention group, minor changes in referral patterns that were actually there, may have not been revealed in our analyses due to power issues. Nonetheless, for breast cancer this study shows that a one point higher score on indicators for medical effectiveness results in a 1.0% (p=0.01) increase in the likeliness of referring a patient to that hospital. Given the differences of a maximum of nine percentages between the best and worst performing hospital on breast cancer (see Additional file 1: Table S1), the total effect may sum up to a total of 9%. This finding is in line with the qualitative statements of the GPs, where they mentioned that the breast cancer indicators for medical effectiveness appeared to be the most valid, reliable and differentiating indicators and therefore the most convincing indicators to alter their referral patterns.

The finding that GPs rely for their referrals on other aspects than quality information is in line with previous survey studies, that show that GPs usually refer patients to hospitals based on their (informed) opinion about a particular hospital rather than quality information [26,27]. To improve our health care system, meaningful outcome measures should be developed and published in a comprehensible way for GPs and patients. For many conditions, at least in the Dutch context, outcome indicators are available via administrative data, clinical registries or Patient Reported Outcomes and should become public.

While our experimental design is robust due to the randomization and the use of an internal and external control group and also comparing the intervention group with the referral patterns in the previous year, our study may have suffered from possible limitations. First, the number of GPs included in our study is limited, therefore minor changes in referral patterns might not result in statistically significant changes, although we do include a substantial number of referrals in our study. Second, the period of our study is only one year. Therefore results in this study should be interpreted as short term results.

## Conclusion

Our study provides empirical evidence that GP referral patterns were unaffected by the available quality information, except for the outcome indicators for breast cancer care that were presented. This finding was surprising since our study was designed to identify changes in hospital preference (1) amongst the most motivated GP’s, (2) that received personal clarification of the performance indicators, and (3) selected indicators/conditions from a large set of indicators that they believed were most important. This finding may differ when quality information is based on outcome indicators with a clinically relevant difference, as shown by our indicators for breast cancer treatment. We believe that the current set of (largely process) hospital quality indicators do not serve the GP’s information needs and consequently quality plays little role in the selection of hospitals for treatment.

## Endnotes

^a^Estimations of the local GP association are that 95% of GPs use Health Domain.

^b^We ensured that the higher the score the more positive the score was. For e.g. the indicator percentage infections we subtracted this score from 100%.

## Competing interests

The authors declare that they have no competing interests.

## Authors’ contributions

DI drafted the manuscript and contributed to all other aspects of the study. XK was involved in the data analyses and performed critical revision of the manuscript. Both authors read and approved the final manuscript.

## Pre-publication history

The pre-publication history for this paper can be accessed here:

http://www.biomedcentral.com/1471-2296/14/95/prepub

## Supplementary Material

Additional file 1: Table S1 Breast cancer report card. Click here for file

Additional file 2: Table S2 Cataract surgery report card. Click here for file

Additional file 3: Table S3 Hip and knee replacement report card. Click here for file
