# Supplementary material for: The use of quality information by general practitioners: does it alter choices? A randomized clustered study
Source: BMC Fam Pract. 2013 Jul 8;14:95. doi: 10.1186/1471-2296-14-95 (PMC3707858; doi:10.1186/1471-2296-14-95)
Supplement: Additional file 3: Table S3 — Hip and knee replacement report card. [file 1471-2296-14-95-S3.doc]

**Additional file 3: Table S3: Hip and knee replacement report card**


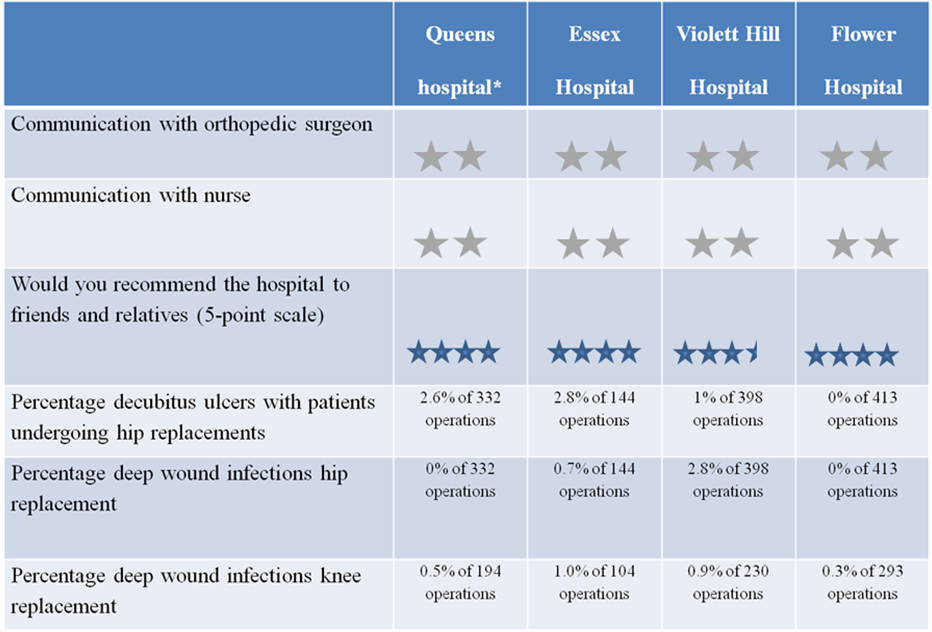


*names of hospitals are feigned (original hospital names were shown at report cards)

stands for statistical significantly lower patient average than national average meaning

stands for average patient experience

stands for statistical significantly higher than national average
